# MIGS as a Simple and Efficient Method for Gene Silencing in Rice

**DOI:** 10.3389/fpls.2018.00662

**Published:** 2018-05-16

**Authors:** Xuelian Zheng, Lijia Yang, Qian Li, Linyi Ji, Aiting Tang, Lili Zang, Kejun Deng, Jianping Zhou, Yong Zhang

**Affiliations:** Department of Biotechnology, School of Life Sciences and Technology, Center for Informational Biology, University of Electronic Science and Technology of China, Chengdu, China

**Keywords:** rice, starch, MIGS, OsGBSS, amylose content

## Abstract

MiRNA-induced gene silencing (MIGS) technology is a special kind of RNA interference technology that uses miR173 to mediate the production of *trans*-acting siRNA (ta-siRNA) to achieve target gene silencing. This technique has successfully mediated the silencing of interested genes in plants such as Arabidopsis, tobacco, petunia, etc. In order to establish the MIGS technology system in monocots such as rice, we constructed the MIGS backbone vectors pZHY930, pZHY931, pZHY932, and pZHY933 with different with promoters to regulate the expression of miR173 and miR173_ts. The rice OsPDS reporter gene was selected to compare the efficiency of four MIGS backbone vectors by the ratio of albino plants. The results showed that all the four backbone vectors could effectively mediate the target gene silencing, and pZHY932 showed highest efficiency up to 90%. Through MIGS silencing of endogenous OsROC5 and OsLZAY1 in rice, we successfully obtained rice mutant plants with rice leaf roll and tillering angles increasing, and further confirmed that MIGS backbone vector can efficiently mediate target gene silencing in rice. On the other hand, in order to verify the efficiency of MIGS-mediated multi-gene silencing in rice, we constructed two double-gene silencing vectors OsPDS and OsROC5, OsPDS and OsLZAY1, based on pZHY932 backbone vector. Double mutant rice plants with increased leaf and albino tiller angles. And we successfully obtained bladed leaf albino seedling and increased tillering angle albino seedling double-silencing mutations. We further constructed a MIGS-OsGBSS gene silencing vector and obtained rice materials with significantly reduced amylose content. This result indicated that MIGS could be an efficient method in monocots gene silencing and gene function analysis.

## Introduction

RNA interference (RNAi) is a highly conserved biological process in which the 20–30 nt small RNA molecules (sRNA) inhibit gene expression by causing the degradation of specific mRNA molecules (Fire et al., 1998). Since its discovery in 1990s, RNAi has been widely used in the research and application of the functional genomics of animals and plants. Endogenous sRNA in living cells is widespread, according to the characteristics of its precursors; we divided sRNA into two main types: microRNA (miRNA) and small interfering RNA (siRNA). Both of them are length 20–25 nt, and produce by Dicer or DCL (Dicer-like) which are similar to RNase III (Agrawal et al., 2003). SiRNAs derive from fully complementary double stranded RNA and have a typical sequence diversity. The miRNAs processes by the pri-miRNA with the hairpin structure as well as typical sequence specificity (Chapman and Carrington, 2007; Xie and Qi, 2008; Voinnet, 2009). There are many kinds of siRNAs, according to the different types of the precursor sequence and the mechanism of formation, they can be divided into several types, include tasiRNA (*trans*-acting siRNA), natsiRNA (natural antisense transcript-derived siRNAs), hcsiRNA (heterochromatic siRNA), rasiRNA (repeat-associated siRNA) and others.

tasiRNA is a special siRNAs in higher plants, which generates from precursor transcripts by the specific miRNA/AGO (ARGONAUTE protein) complexes guided cleavage, and converts to dsRNA, and then processes into short 21 nt long RNA duplexes (Peragine et al., 2004; Vazquez et al., 2004; Axtell et al., 2006). tasiRNA is a combination of miRNA and siRNA silencing pathway, which has the characteristics of both siRNAs and miRNAs. Like siRNAs, tasiRNA undergo similar processing and arise from dsRNA, and their generation are both RDR6 (RNA-dependent RNA polymerase 6) dependent (Vazquez et al., 2004). However, tasiRNA like miRNAs binds with less sequence specificity to their targets, and does not require full sequence complementarity (Peragine et al., 2004; Yoshikawa et al., 2005). In Arabidopsis there are four families of tasiRNA genes (TAS loci), and different miRNAs corresponded to different TAS genes (TAS1, TAS2 corresponding miR173, TAS3 corresponding miR390, and TAS4 corresponding miR828) (Chapman and Carrington, 2007). TAS1 and TAS2 transcripts undergo miR173-guided cleavage by AGO1 at the 5′ end, dsRNA synthesis by RDR6, and generates the 21-nt siRNA by DCL4 to target complementary mRNAs. The TAS4 family is similar to that of TAS1 and TAS2, except for the guided miR828. Compared with those single mRNA binding family, TAS3 transcripts require the guide miR390 bind the transcript at two sites, and then cleave at the 3′ binding site only by AGO7 (Gasciolli et al., 2005; Axtell et al., 2006; Nakazawa et al., 2007; Martínez de Alba et al., 2013).

MIGS (miRNA-induced gene silencing) is an efficient technique to induce gene silencing in Arabidopsis, tobacco, soybean, and petunia (Felippes et al., 2012; Yao et al., 2015; Jacobs et al., 2016). By fusing a 22 nt long miR173 target site (miR173_ts) to the 5′ end of the target gene fragment, we constructed the MIGS vector through simply PCR reaction. Since the guide miR173 can incorporate into the same MIGS vector, the co-expression of guide miR173 and miR173_ts-target fragment lead MIGS can be used in any plant species. MIGS can also be used in multiple-gene silencing with a single vector by directly link the miR173_ts to different target fragments (Felippes et al., 2012). Compared with other gene silencing methods, VIGS requires specific viral vectors, which limited to a few hosts. In hpRNAi, the inserted gene fragment needs to be cloned as an inverted repeat sequences. amiRNA designing usually requires multiple PCR reactions to replace mature miRNA in precursor backbone. MIGS offers an effective and more convenient sRNA silencing approach than hpRNA, amiNRA and VIGS. However, there is no research investigate the efficiency mechanisms in monocot plants.

Here, we provide an efficient alternative to monocot gene silencing strategies. Based on tasiRNA MIGS principle and monocot specific expression cascade, we constructed three different monocot MIGS backbones. By using rice endogenous gene OsPDS (Rice phytoene desaturase precursor, GenBank Accession No. AF049356) as visible reporter, we evaluated the efficiency of different backbones, and obtained OsPDS, OsROC5 (Rice outermost cell-specific 5 gene, GenBank Accession No. AB101648), OsLAZY1 (Rice shoot gravitropism gene, GenBank Accession No. DQ855268) silencing rice mutants through monocot MIGS system. Meanwhile, we detected the monocot multiple MIGS system by achieving the OsPDS-OsROC5 and OsPDS-OsLAZY1 dual mutant rice. Furthermore, we successfully knocked down the rice OsGBSS (Rice granule bound starch synthase 1 gene, GenBank Accession No. FJ235787) gene expression by MIGS technology, and obtained the transgenic rice less seeds with significant decrease in amylose content. These indicate that MIGS technology has a wide range of application prospects in rice research and breeding.

## Materials and Methods

### Plant Material and Growth Condition

*Oryza sativa* Japonica Group cultivar Nipponbare was used as the wild type and transformation host. Matured seeds were geminated for 2 days at 32°C in the dark. Germinated seeds were then transferred to soil, and seedlings were grown under 16 h light at 30°C 8 h dark at 22°C in greenhouse.

### Plasmid Construction

**FIGURE 1:**
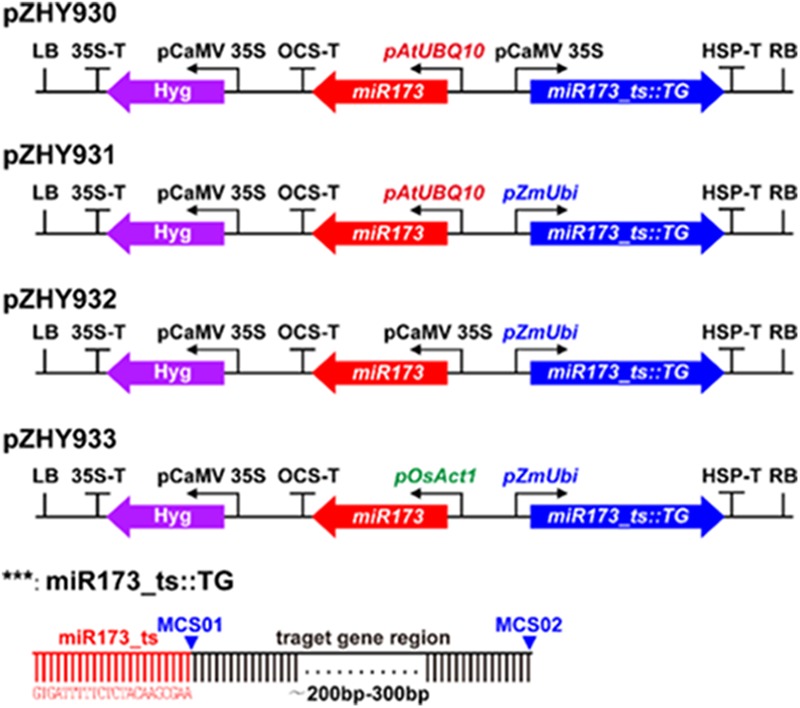
MiRNA-induced gene silencing (MIGS) backbone vectors containing different promoter combination to evaluate efficiency of gene silencing in rice. In order to obtain plant MIGS system for monocycle plant such as rice, we constructed four different MIGS backbone vectors. In pZHY930 backbone, the expression of miR173 and miR73-ts sequences were consistent with that in Felippes et al. (2012) except that the resistance selection gene was changed to hygromycin. Compared with pZHY930 vectors, the pZHY931, pZHY932, and pZHY933 backbones have ZmUbi, AtUbq10 and OsAct promoters to induce the highly expression of miR173 and miR173_ts sequence, respectively. The 200–300 bp target gene fragment was placed downstream of miR173-ts sequence, and the reporter gene MIGS expression vectors with different backbones were obtained. LB, left board; RB, right board; pAtUBQ10, Arabidopsis ubiquitin 10 gene promoter; pCaMV35S, CaMV 35S promoter; pZmUbi, corn Ubiquitin 1 gene promoter; pOsAct1, rice actin gene promoter; miR173, Arabidopsis miR173 expression sequence; miR173_ts, miR173 target sequence; Hyg, hygromycin resistant marker gene; HSP-T, HSP terminator; OCS-T, octopine synthetic enzyme gene terminator; 35S-T, CaMV 35S terminator; MCS01, MCS02, multiple cloning sites, for insertion into a sequence of target gene interference region.

Construction of MIGS backbone vector pZHY930 based on binary expression vector MIGS 2.1 (Felippes et al., 2012). The artificial miR173 sequence place between AtUBQ 10 promoter and OCS terminator by fusion PCR, correspondingly the synthetic miR173_ts sequence place between the CaMV 35S promoter and the HSP terminator using fusion PCR. The MCS (KpnI-SmaI double RE sites) is downstream of the miR173_ts sequence to insert the target gene 200–300 bp fragment. A similar method substitute for the ZmUbi, OsACT promoter to obtain pZHY931, pZHY932 and pZHY933 backbone vectors (**Figure 1**).

According to OsPDS gene sequence, the total RNA of Nipponbare leaves extracts from TRIzol (Invitrogen, United States) method uses as template, ZLL008F (5′-GTGATTTTTCTCTACAAGCG AAGGTACCATTTCTTCAGGAGAAGCATGGTTCTAAGATG-3′) and ZLL008R (5′-TTTCATCTTCATCTTCATATCTCGAGCCCGGGATCAACTGGTGTTGCAAAAACATAAGC-3′) uses as primers to amplify 289 bp OsPDS gene fragment by RT-PCR. The amplified OsPDS gene fragment introduced into the KpnI-SmaI digested pZHY930 vector to obtain the MIGS expression vector pZHY930::OsPDS. The pZHY931::OsPDS, pZHY932::OsPDS, pZHY933::OsPDS vectors were constructed by similar method. The pZHY930::OsROC5, pZHY931::OsROC5, pZHY932::OsROC5, and pZHY933::OsROC5 vectors were constructed by primers ZLL026F (5′-GTGATTTTTCTCTACAAGCGAAGGTACCCGCTGGCGCTGGTTCTA GCGCGGGC-3′) and ZLL026R (5′-TTTCATCTTCATCTTCATATCTCGAGCCCGGGCTTCAT CTGCGTGCGGCGATTCTGG-3′). The pZHY930::OsLAZY1, pZHY931::OsLAZY1, pZHY932::OsLAZY15, and pZHY933::OsLAZY1 vectors were constructed by primers ZLL027F (5′-GTGATTTTTCTCTACAAGCGAAGGTA CCATCCTTCAAATCTTCCACAGGAAAG-3′) and ZLL027R (5′-TTTCATCTTCATCTTCATATCTCGAGCCCGGGAGTCGGCATCAGTCTTGATCCAGT-3′). The amplified OsGBSS gene fragment (ZLL014F: 5′-GTGATTTTTCTCTACAAGCG AAGGTACCATGTCGGCTCTCACCACGTCCCAGCTCGCC-3′, ZLL014R: 5′-CCAAATATTTC ATCTTCATCTTCATATCTCGAGCCCGGGAGCCATGGCAGGGGGGAGGC-3′) introduced into the KpnI-SmaI digested pZHY932 vector to obtain the MIGS expression vector pZHY932::OsGBSS (**Figure 5A**).

In order to obtain the double gene MIGS vectors pZHY932::OsPDS::OsROC5 (**Figure 4A**) and pZHY932::OsPDS::OsLAZY1 (**Figure 4B**), the 5′ ends of the two gene fragments were fused with a miR173_ts sequence, respectively, then OsPDS-OsROC5 gene fragment (ZLL028F: 5′-TTTGCAACACCAGTTGATGTGATTTTTCTCTACAAGCGAACGCTGGCGCTGGTTCTAG-3′, ZLL028R: 5′-CTA GAACCAGCGCCAGCGTTCGCTTGTAGAGAAAAATCACATCAACTGGTG TTGCAAA-3′), OsPDS-OSLAZY1 gene fragment (ZLL029F: 5′-TTTGCAACACCAGTTGATGT GATTTTTCTCTACAAGCGAACGCTGGCGCTGGTTCTAG-3′, ZLL029R: 5′-CTAGAACCAGCGCCAGCGTTCGCTTGTAGAGAAAAATCACATCAACTGGTGTTGCAAA-3′) with miR173_ts were fused to pZHY932 backbone.

### Agrobacterium-Mediated Rice Transformation and Transgenic Positive Rice Detection

Above expression vectors were transferred into *Agrobacterium tumefaciens* EHA105, respectively. Agrobacterium-mediated rice transformation was based on a previous method (Hiei et al., 1994; Tang et al., 2017). At the same time, pCambia1301 was transferred into Nipponbare rice used as negative control. Dehusked seeds sterilize and culture on solid medium at 28°C in a dark growth chamber for 2–3 weeks. Actively growing calli were Incubated in an Agrobacterium suspension for 30 min and transferred onto screening medium containing 50 mg/L hygromycin and 500 mg/L Carbenicillin. During the screening stage, hygromycin resistant calli transferred onto fresh screening medium every 2 weeks. After 6 weeks, growing calli transferred into regeneration medium for 4 weeks. The hygromycin resistant seedlings then transferred into fresh solid medium and grown for 2 weeks before planted into soil. To detect transgenic positive rice plants, PCR performed using genomic DNA isolated from transformed rice leaves by CTAB DNA extraction method.

### RNA Extraction and Quantitative RT-PCR

The total RNA was extracted from transgenic rice leaves (for OsPDS, OsROC5, OsLAZY1, and OsActin gene detection) or endosperm (for OsGBSS gene detection) at the same development time by TRIzol method, and reverse transcription was carried out by RevertAid First Strand cDNA Synthesis Kit (Thermo Scientific, United States). RT-PCR was performed with PCR master mix kit (Tiangen, China) through the manufacturer’s instructions. OsActin (OsActin-F: 5′-CCTT GATTATGAGCAGGAGCTG-3′, OsActin-R: 5′-AAGTGATCTCCTTGCTCATAC-3′) fragment was used as an internal control to detect differences in expression levels of target gene in wild type and transgenic plants. Three biological replicates (three random-selected independent transgenic lines for each transgenic plants with different vectors) were examined to ensure reproducibility and each experiment was performed three times independently.

### Iodine Staining of Rice Endosperm and Microscope Observation

The method used to rice starch granules iodine staining was performed according to Nakamura’s (Nakamura et al., 1995). Harvested rice seeds were husked, each strain was randomly selected for eight seeds to immerse in water for 24 h cut the soaked seeds horizontally with sharp blades. Absorb the prepared iodine solution to endosperm staining. After 2 min, remove the excess dye with a clean filter paper to ensure that the reaction time of the transgenic lines and the control is same. For microscopic observation, a little endosperm tissue of soaked seeds took with a tweezers to place on the slide. Straightly pressed with coverslips. A little bit of iodine staining was added to the end of the coverslip for staining. After 2 min, remove excess dye from the other end with a clean filter paper. For SEM observation, rice seeds were fractured across the short axis and dried completely under low pressure. The surface was sputter-coated with gold and observed by using a JMS-7500 scanning electron microscope at 5.0 KV (Li et al., 2005).

### Measurement of Starch Content and Amylose Content in the Endosperm

Determination of total starch and soluble sugar by anthrone sulfuric acid method (Yemm and Willis, 1954; Li et al., 2011). Harvested rice grains were air-dry before analysis. Some dried kernels were selected, peel the seed coat, and everything that remained was dried together to constant weight at 60°C before the weight was determined. Then the dried endosperm was ground to powder for later study. Samples of the powdered endosperm prepared as mentioned above (50 mg) were extracted with 4 mL of 80% ethanol at 80°C for 40 min, followed by two extractions with 2 mL of 80% ethanol. The remaining pellets were dried at 60°C to remove the ethanol, and boiled for 10 min with 3 mL double-distilled water in 50 mL centrifuge tubes. Then the samples were cooled to room temperature, and 4 mL HClO_4_ was added to decompose the starch. Starch in the paste hydrolyzed for 15 min, and the amount of soluble sugar was determined with the anthrone reagent using glucose as the standard. Starch content calculating used the formula: Starch content =G ^∗^0.9/DW ^∗^100%.

For amylose content detection, Milled samples (50 mg per sample) added 10 ml 1 mol/L KOH solution heated on the boiling water bath for 30 min. The amylose content was performed according to the procedure described in Hovenkamp-Hermelink et al. (1988), Zhu et al. (2008). For all the data analysis, three individual transgenic lines were performed and three individual plant sample were selected for each line. Each sample was tested for three times. The data were analyzed by Excel and SPSS.13 for calculation and significant differences analysis.

## Results

### Monocot MIGS Vectors Based on Different Backbone

In order to make MIGS system work well in rice, we designed four different MIGS backbones and constructed according to MIGS vectors in Arabidopsis (Felippes et al., 2012). The MIGS pZHY930 backbone has a miR173_ts sequence followed by a multiple cloning site (MCS) for insertion of a fragment of target gene by sub-cloning, which between the CaMV 35S promoter and HSP terminator. An expression cassette with the miR173 precursor placed between the Arabidopsis UBQ10 constitutive promoters (AtUBQ10) and constructed OCT terminator into pZHY930, to ensure highly constitutive expression of miR173. The pZHY931 pZHY932 and pZHY933 backbones have different monocot constitutive promoters to maintain high expression rate of miR173 and miR173_ts. In pZHY931, the maize UBI constitutive promoter (ZmUBI) replaced the CaMV 35S promoter in pZHY930 to drive the expression of miR173_ts and target gene fragment. Comparing with pZHY931, the pZHY932 vector includes similar expression cassettes while the CaMV 35S promoter is in place of the Arabidopsis UBQ10 constitutive promoter. Furthermore, in pZHY933 we replaced CaMV 35S ZmUBI promoter to control expression of miR173_ts and target gene, while the rice actin promoter (OsACT) replaced AtUBQ10 to regulate the guide miR173 expressing. All these backbone vectors include an expression cassette with hygromycin resistance genes in plants (**Figure 1**).

### Efficiency of OsPDS Gene Silencing by Using Different MIGS Vectors in Rice

**FIGURE 2:**
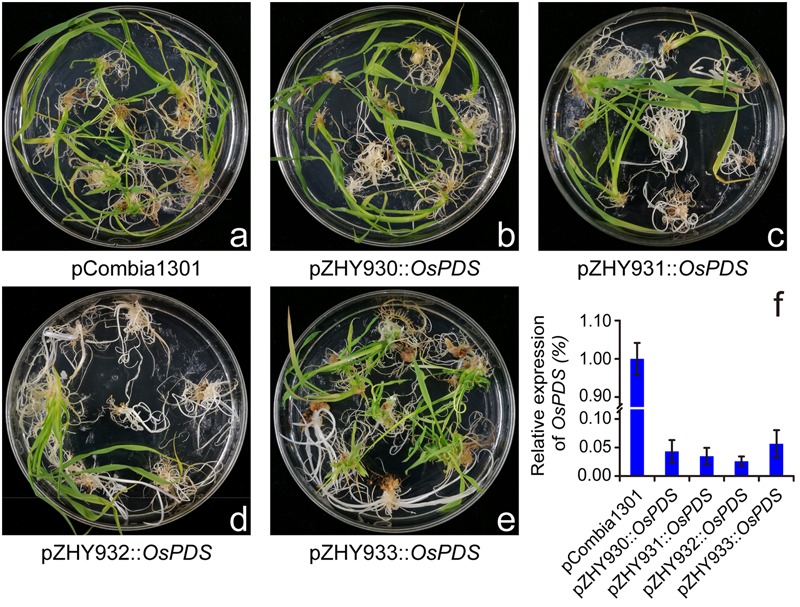
Comparison of MIGS effect of different backbones on rice endogenesis PDS gene. MIGS induced down-regulation of OsPDS gene lead to albino phenotype. Comparing with pCambia1301 **(a)** control plants showed normal green, pZHY930::OsPDS **(b)**, pZHY931::OsPDS **(c)**, pZHY932::OsPDS **(d)**, and pZHY933::OsPDS **(e)** MIGS plants showed different albino efficiency. The transgenic rice lines with pZHY932 backbone were almost albino and had the highest efficiency of gene silencing. **(f)** Semi-quantitative RT-PCR results showed that all the MIGS vectors could effectively reduce the expression level of endogenous OsPDS gene. Error bars indicate standard deviation (*n* = 3 independent experiments).

For investigating the efficiency of different monocot backbones, we selected OsPDS gene as a target gene to construct the different MIGS vectors corresponding to pZHY930, pZHY931, pZHY932, and pZHY933 backbones, respectively. Four MIGS vectors with OsPDS target gene and pCambia1301 control vector were transformed into rice variety Nipponbare, respectively, regeneration transgenic rice plantlets were obtained from hygromycin resistant calli. Comparing normal green plantlets from pCambia1301 control vector, all the MIGS vectors regenerated PDS gene-silencing albino plantlets. It indicated that MIGS system functions effectively in rice (**Figures 2b–e**).

As expected, different MIGS backbones showed obviously different efficiency in OsPDS gene-silencing according to the proportion of white plantlets in all transgenic plantlets (albino rate), while pCambia1301 control plants were all green. As it was shown in **Figure 2** and **Table 1**, albino rate of pZHY930::OsPDS backbone was about 10%, and the pZHY931::OsPDS and pZHY933::OsPD of is about 40%, and albino rate of pZHY932::OsPDS was dramatically increased to over 90%.

**Table 1:** Comparison of miRNA-induced gene silencing (MIGS) effect on different backbones.

Targeted locus	MIGS backbone	Number of PCR positive seedlings	Number of targeted locus with modified phenotype	MIGS efficiency
OsPDS	pZHY930	64	8	12.50%
	pZHY931	51	15	29.41%
	pZHY932	63	57	90.48%
	pZHY933	58	23	39.66%
OsROC5	pZHY930	36	3	8.30%
	pZHY931	36	7	19.40%
	pZHY932	42	37	88.10%
	pZHY933	39	10	25.64%
OsLAZY1	pZHY930	38	4	10.53%
	pZHY931	37	9	24.32%
	pZHY932	38	32	84.21%
	pZHY933	40	11	27.50%

Further results of RT-PCR and semi quantitative analysis shows that the OsPDS mRNA level of pZHY930, pZHY931, pZHY932, and pZHY933 transgenic plants were significantly reduced, compared with that of pCambia1301 plants (**Figure 2f**), which consistent with the phenotype results. The ideal PDS gene-silencing phenotype and effectively inhibition of OsPDS transcriptional level exactly indicates that monocot MIGS vectors function well in rice.

### ROC5 and LAZY1 Gene Silencing Using Monocot MIGS System in Rice

**FIGURE 3:**
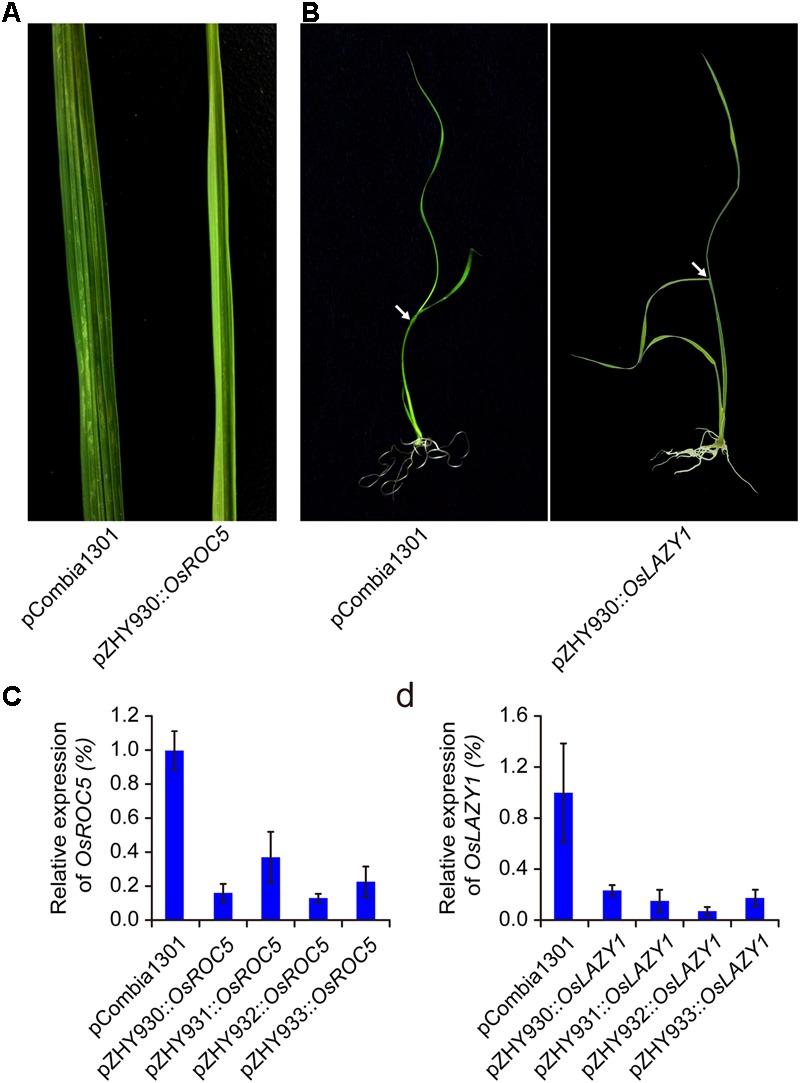
Comparison of MIGS effect on rice endogenesis ROC5 and LAZY1 genes silencing. **(A)** MIGS-OsROC5 gene down-regulated silencing rice plants showed roll leaf phenotype comparing the pCambia1301 plants. **(B)** MIGS-OsLAZY1 gene down-regulated silencing rice plants showed increased branching angle (arrow) phenotype. **(C,D)** Semi-quantitative RT-PCR showed that OsROC5 and OsLAZY1 gene expression levels were significantly decreased in MIGS silencing plants with pZHY930, pZHY931, pZHY932, and pZHY933 backbones. Error bars indicate standard deviation (*n* = 3 independent experiments).

For further investigating the widely usage and high efficiency of monocot MIGS vectors, OsROC5 and OsLAZY1 genes controlling leaf rolling and tiller-spreading phenotype were selected to construct MIGS silencing vectors based on pZHY930, pZHY931, pZHY932, and pZHY933 backbones (Li et al., 2007; Takeshi and Moritoshi, 2007; Zou et al., 2011). All the MIGS vectors introduced into wild type Nipponbare rice via *A. tumefaciens-*mediated transformation, while the pCambia1301 vector used as control. All MIGS-OsROC5 transgenic lines showed typical leaf-rolling traits in different efficiency. The mature leaves of OsROC5 gene silencing lines were outcurve rolled, whereas the pCambia1301 control leaves remained flat (**Figure 3A**). Furthermore, by RT-PCR of total RNA extracted from leaves of each transgenic line in five-leaf stage, OsROC5 mRNA expression levels were dramatically lower in MIGS lines than in the pCambia1301 control (**Figure 3C**). Similarly MIGS-OsLAZY1 plantlets displayed larger tiller angles and spread-out plant architecture compared to the control plants (**Figure 3B**), and the transcriptional level of MIGS-OsLAZY1 plantlets were significantly lower than the control plants (**Figure 3D**).

Different silencing efficacy of OsROC5 and OsLAZY1 genes in pZHY930, pZHY931, pZHY932, and pZHY933 vectors were similar to MIGS-OsPDS vectors, and was shown in **Table 1**. The pZHY930 backbone was the least efficient vector for MIGS gene silencing in monocot plants. And the pZHY932 backbone with CaMV 35S promoter controlling expression of the guide miR173 unit, combining ZmUbi promoter controlling expression of miR173_ts and target gene interference region, was the most efficient backbone in monocot plant, with efficiencies of around 90% (90.48% in OsPDS, 88.10% in OsROC5, 84.21% in OsLAZY1).

### Multiple Gene Silencing Using Monocot MIGS in Rice

**FIGURE 4:**
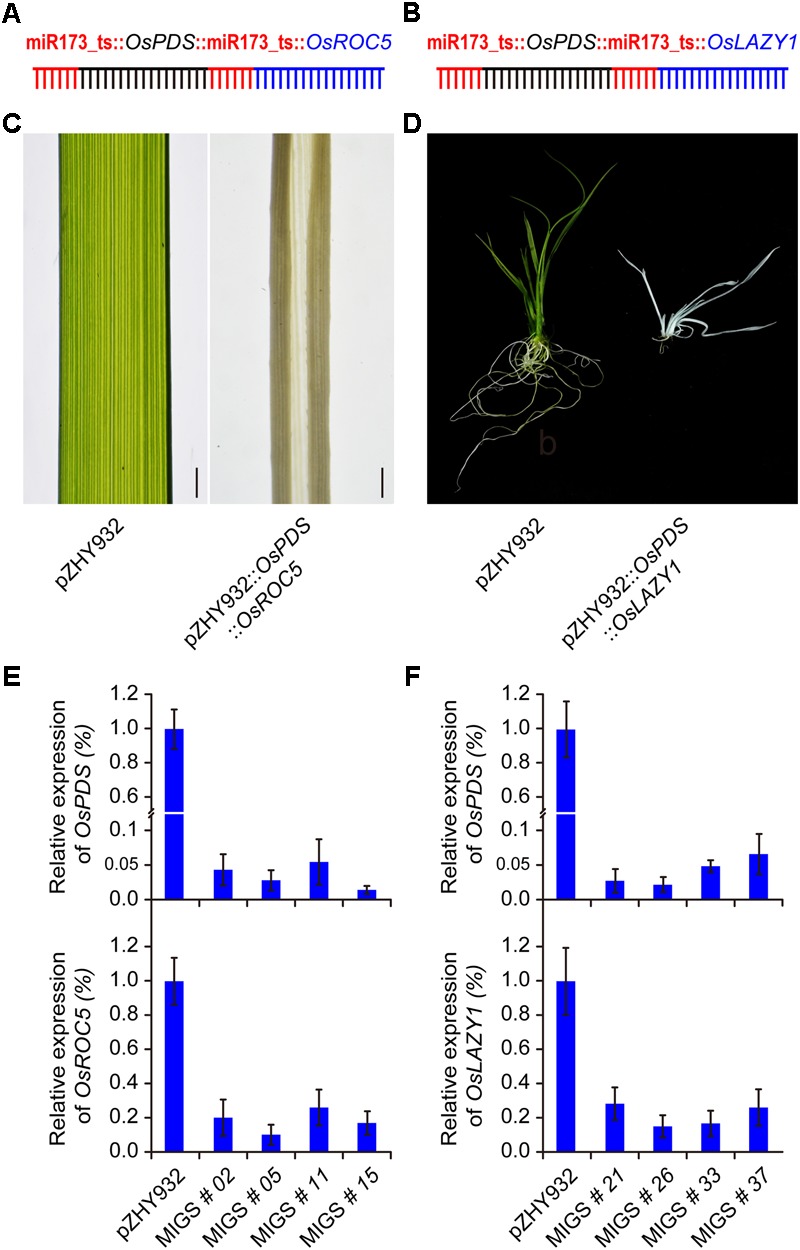
MiRNA-induced gene silencing induced multiple gene silencing in rice. **(A,B)** Schematic description of OsPDS and OsROC5 gene simultaneously down-regulated MIGS vector, OsPDS and OsLAZY1 simultaneously down-regulated MIGS vector were constructed with pZHY932 backbone, respectively. **(C,D)** OsPDS and OsROC5 gene simultaneously down-regulated rice plants showed albino and roll leaf phenotype, while OsPDS and OsLAZY1 gene simultaneously down-regulated rice plants showed albino and increased branching angle phenotype. **(E,F)** Semi-quantitative RT-PCR results showed that OsPDS, OsROC5, and OsLAZY1 gene expression levels were significantly decreased in all MIGS silencing plants. Error bars indicate standard deviation (*n* = 3 independent experiments).

MiRNA-induced gene silencing could be used to simultaneously silence two different genes in Arabidopsis (Felippes et al., 2012). We detected the multiple gene silencing effect on monocot MIGS system based on highest efficient backbone pZHY932. For dual gene silencing, two miR173_ts sequences followed by two different target gene fragments OsPDS-OsROC5 and OsPDS-OsLAZY1, respectively, were fused into a whole expression cassette, driven by ZmUBI promoter in pZHY932 (**Figures 4A,B**). Dual MIGS vectors were transformed into wild type Nipponbare and pZHY932 backbone vector used as control. Regeneration transgenic plantlets were confirmed by PCR detection. Expectedly, the transgenic plantlets of pZHY932::OsPDS::OsROC5 showed albino plant and outcurve rolling leaf at the same time, while the pZHY932 plants had green and flat leaves (**Figure 4C**). The pZHY932::OsPDS::OsLAZY1 plants displayed albino and spread-out phenotype as expected, and the pZHY932 control was normal (**Figure 4D**). RT-PCR results demonstrated that the OsPDS and OsROC5 genes expression level was significantly decrease simultaneously, and the similar results were obtained in OsPDS and OsLAZY1 dual gene silencing plants (**Figures 4E,F**). These results suggested that the monocot MIGS system successfully inhibit the double gene expression simultaneously in rice, and it could use not only in single gene silencing but also in multiple gene silencing in monocot plants.

### MIGS Silencing of OsGBSS Gene Affected Amylose Content in Rice Seeds

**FIGURE 5:**
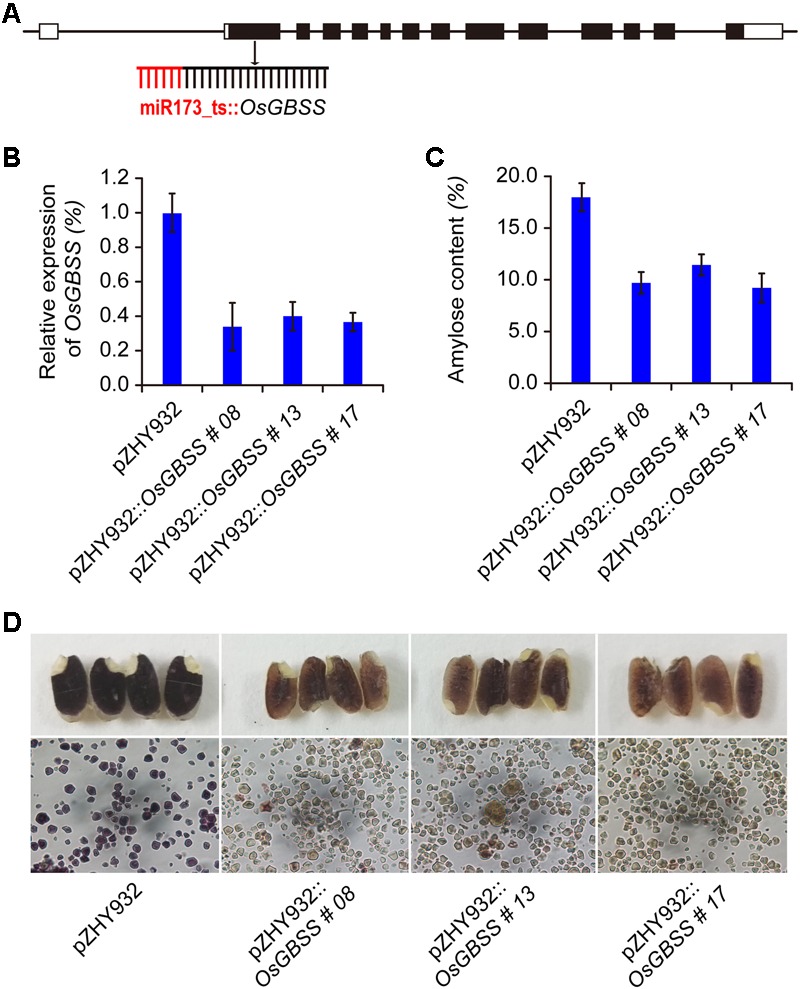
Application of MIGS strategy to modify starch composition in rice seed. **(A)** The OsGBSS gene target site was shown in schematic, MIGS-OsGBSS vector was constructed with the highest efficiency backbone pZHY932. **(B)** Semi-quantitative RT-PCR results showed that OsGBSS gene expression level of rice endosperm were decreased in MIGS silencing rice lines. Error bars indicate standard deviation (*n* = 3 independent experiments). **(C)** The amylose content of the pZHY932::OsGBSS transgenic seeds were decreased significantly, error bars indicate standard deviation (*p* < 0.05, *n* = 3 independent experiments). **(D)** The iodine staining results of mature seeds of MIGS-OsGBSS silencing plants showed brown red, and the starch granules of pZHY932::OsGBSS showed light brown in optical microscopy. While the corresponding pZHY932 control seeds were dark-blue, and the starch granules were blue-purple.

As mentioned above, MIGS could mediate effective gene silencing of rice endogenous genes. We used MIGS technology to silence OsGBSS gene which regulated the synthesis of amylose content in rice to obtain low amylose rice seeds. The MIGS-OsGBSS vector pZHY932::OsGBSS was constructed based on the most efficient backbone pZHY932 and the first exon of the OsGBSS gene was used as the target fragment (**Figure 5A**). More than 50 transgenic rice lines were obtained by *A. tumefaciens* mediated transformation. Five transgenic positive lines performed by genomic PCR and the agronomic traits of these transgenic lines showed no difference from that of pZHY932 control. Three transgenic lines were selected for subsequent experiments. The expression of OsGBSS gene in the endosperm tissue of three lines were decreased significantly (**Figure 5B**). The results of iodine-stained showed that there was a significant difference in the color of endosperm between control and MIGS-OsGBSS seeds. The wild-type control showed typical blue-violet, while the seeds of MIGS-OsGBSS transgenic lines showed brown red or light brown (**Figure 5D**). The observation of iodine-stained starch granules by optical microscopy showed that the starch granules of control and gene silencing lines were polygonal. The morphological size was not significantly different, while the control starch granules were blue–purple, indicating that the amylose ratio was higher, while the gene silencing strain of starch granules was light brown, indicating that mainly containing amylopectin (**Figure 5D**). At the same time, the SEM was performed to observe whether the amylose content change had an effect on the structure of the starch granules by JMS-7500 SEM. The results showed that the starch granules were smooth and irregular in the MIGS gene silencing lines and wild type, there was no obvious difference (Data not shown).

Furthermore, the amylose content and amylopectin content of T1 generation seeds were quantitatively determined by dual wavelength method (Zhu et al., 2008). The results showed that the amylose content of the three gene silencing lines decreased significantly compared with the control seed. The amylose content of transgenic lines pZHY932::OsGBSS-08, pZHY932::OsGBSS-13, and pZHY932::OsGBSS-17 were 10.4, 12.0, and 9.9%, respectively, which were significantly lower than those of control 18.1% (**Figure 5C**). Thus, there was no significant difference in total starch content between the transgenic lines and controls (data not show). The above results indicated that the pZHY932::OsGBSS vector was effective in eliciting the MIGS silencing of the rice-derived GBSS gene and thus the decrease of the amylose ratio in the seed.

These results indicated that the MIGS system established in monocotyledonous plants had been successfully applied to the regulation of rice starch synthesis pathway, not only for the directional silencing of granulated starch synthase gene, but also down-regulated amylose content in the endosperm of T1 generation. This technology provided a feasible solution for rice breeding and improved starch quality.

## Discussion

MiRNA-induced gene silencing is a very efficient and simple gene silencing technique. As long as the 22nt miR173_ts target site linked to the gene-silenced target fragment, the guide miR173 is sufficient to trigger the *trans*-acting siRNA reaction, which in turn causes the silencing of the target gene. On the other hand, the size of the target gene fragment used in MIGS is about 200–500 nt, and the size of the fragment can effectively induce the silencing of the target gene, which makes the construction of MIGS vector very simple and requires only one step of PCR reaction achieve. In addition, MIGS can not only silence multiple homologous genes, but also very simply silence multiple different genes at the same time. It is only need to connect multiple MiR173_ts sequences to fragments of different target genes, respectively. Thus MIGS has been successfully used in tobacco, Arabidopsis, *Medicago truncatula*, soybean, petunia, and other plants for gene silencing (Felippes et al., 2012; Benstein et al., 2013; Imin et al., 2013; Yao et al., 2015; Jacobs et al., 2016). Fellipes first studied miR173-mediated MIGS in *Arabidopsis thaliana* and tobacco, and MIGS can effectively induce single gene, double gene silencing in Arabidopsis and tobacco (Felippes et al., 2012). Benstein used MIGS to silence the phosphoglycerate dehydrogenase I (PGDH1) gene in *A. thaliana*, and the transgenic positive plants showed significant growth inhibition (Benstein et al., 2013). In addition, the C-terminal protein 1 (CEP1) gene of *M. truncatula* was successfully silenced by the MIGS2.1 vector (Imin et al., 2013). It has also been reported that the expression of CHS (chalcone synthase) and PDS (phytoene desaturase) gene was successfully inhibited by the MIGS technique in petunia, and the white albino plants were obtained (Vazquez et al., 2004; Yao et al., 2015).

We successfully established a simple and efficient MIGS system for single and multiple gene silencing in monocot plant rice. We constructed four different backbone vectors pZHY930, pZHY931, pZHY932, and pZHY933 by altering the promoters of the control guide miR173 and miR173_ts sequences, and the efficiency of induction of gene silencing in four backbone vectors had been compared and evaluated by using OsPDS, OsROC5, and OsLAZY1 genes as reporter genes. After comparing the data, we found that most of the PCR-positive plantlets of pZHY930 appeared normal green phenotype like control plants, miR173_ts transcription pattern in pZHY930 was not suitable for monocot expression in MIGS technique. In pZHY931 backbone, replacement of original CaMV 35S promoter of miR173_ts with monocot maize ZmUbi promoter, MIGS gene silencing rate increased significantly, which suggested that the monocot constitutive promoter was apparently more suitable for the miR173_ts and target gene expression in rice than the dicot promoter. In pZHY932 backbone, the controlling expression of guide miR173 was replaced by strong constitutive promoter CaMV 35S, which guaranteed the expression of miR173 was significantly increased and the gene-silencing effect was dramatically raised to 90%. It indicated that increasing the expression of guide miR173 by strong promoter 35S might be critical to improving gene silencing efficiency. However, it was unexpected that the pZHY933 backbone vector which had the monocot constitutive promoter OsACT to manipulate the guide miR173 expressing had not shown highly gene silencing efficiency as expected. The reason remained unclear and needed to further experimental analysis.

In addition to visible reporter gene OsPDS, we also successfully silenced the endogenous OsRoc5 and OsLAZY1 genes in rice, and obtained the corresponding gene mutant phenotype. We also successfully silenced the endogenous Roc5 and LAZY1 genes of rice, and all of them obtained the corresponding gene defect phenotype. The first phenotypes of outcurve leaf (oul1) found in T-DNA insertion mutant in OsROC5 gene. Roc5 leaves slightly spiraled at the five-leaf stage and gradually curved toward the abaxial surface. Roc5 contains nine exons and eight introns, and the T-DNA was inserted into the sixth intron in knockout of ROC5 (Zou et al., 2011). In four MIGS-ROC5 vectors, the 319 bp target gene fragment was include all the seventh exon and partial eighth exon according to the full sequence of ROC5 gene. ROC5 gene expression was successfully interference, and MIGS-ROC5 gene silencing plantlets showing abaxial leaf rolling like the ROC5 knockout mutant. LAZY1 gene plays a negative role in polar auxin transport (PAT), which regulated shoot gravitropism by controlling the rice tiller angle. LAZY1 mutant rice enhanced PAT greatly and thus alters the endogenous IAA distribution in shoots, leading to the reduced gravitropism, and therefore the tiller-spreading phenotype of rice plants (Li et al., 2007). The MIGS-LAZY1 plantlets had a larger branching angle, as expected. This demonstrated that the MIGS system can come to universal usage in rice.

The advantage of MIGS technology was that it was easier to co-silence multiple genes simultaneously, and convenient to construct a multi-gene silencing vector only by one-step fusion PCR. In this study, we examined the co-silencing of OsPDS and OsROC5, OsPDS, and OsLZAY1 genes using the most efficient backbone vector pZHY932. We had successfully obtained albino and leaf-rolling plants, as well as plants with increased branching angles and white leaves. The pZHY932 MIGS backbone provided a new experimental program for multiple gene silencing.

Since MIGS technology could achieve the silence of single gene and multiply gene in rice, we applied it to study the quality improvement of rice starch. The composition of rice starch is amylose and amylopectin, and the ratio of amylose and amylopectin is an important research direction of rice breeding. Granular bound starch synthase (GBSS) is encoded by waxy gene (Wx), which mainly controls the synthesis of amylose in rice endosperm (Craig et al., 1998; Nakamura et al., 1998; Geigenberger et al., 2005; Obana et al., 2006; Seferoglu et al., 2014). In 1990, Wang cloned rice Wx gene and proved its function in starch synthesis (Edwards et al., 1999). Takeda used antisense RNA to inhibit the expression of Wx gene and reduced the activity of GBSS, resulting in a significant reduction in amylose content in rice seeds (Baba et al., 1993). Therefore, it was possible to change the starch composition of rice by silencing GBSS gene and reducing the amount of amylose in rice endosperm by MIGS technology. In this study, we selected the pZHY932 backbone with the highest silencing efficiency to construct the OsGBSS MIGS vector. The pZHY932::OsGBSS transgenic positive lines successfully reduced the expression of GBSS in plants. The amylose content in the transgenic seed endosperm decreased from 24.53 to 16.38%, the down-regulation proportion reached 33.2%, which was significantly different from that in the control. It indicated that MIGS technology had effectively achieved the down-regulation of amylose content in rice grains. Interestingly, although MIGS causes OsGBSS gene silencing and amylose content decrease, there was no significant effect on total starch content. The decreased amylose content might compensate by the increased amylopectin, which suggested that the synthesis network of rice starch was complex, it could be moderately regulated.

Compared with the efficiency of single gene silencing (60–95%), MIGS-induced OsGBSS gene down-regulation efficiency was lower than that of OsPDS, OsROC5, and OsLAZY1 genes. This should relate to the selection of MIGS interfering target sites, and the specificity of target gene fragments. There were also significant differences in the silencing efficiency of different species and different tasiRNA target sites. Generally, target sites with high sequence homology and specificity are more efficient for interfering certain gene. In addition, because of the large number of genes controlling starch traits, the complex metabolic regulation network, and the complementary function of gene family members, the effect of regulating a single gene of the starch synthesis pathway was often more complex than expected. This suggests that we could co-silence multiple genes in the starch synthesis pathway in further studies and regulate the starch composition more conveniently. MIGS could also combine with other co-expression techniques, such as gene stacking technology, to achieve precise regulation of gene expression in monocotyledonous plants.

## Conclusion

RNAi as a highly efficient and specific technical method was widely used in animal and plant genomic function research. There were many types of RNAi technology; different RNA-mediated gene silencing efficiency was also different. MIGS was the interference technology by miRNA-mediated, tasiRNA binding degradation of the target gene that had emerged in plants in recent years. It had a simple construction steps and high efficiency in gene silence. It could simultaneously silence multiple unrelated genes or the same metabolic pathway of different regulatory genes to study the regulation of specific gene function in the organism. In the presence of effective tasiRNA, targeted gene silencing could be achieved in any species.

By changing different monocotyledon promoters to control expression of guide miR173 and miR173-ts sequences, we successfully constructed a backbone vector pZHY932 and MIGS system for efficient gene silencing in monocotyledonous plant rice. The single gene or double gene silencing of different rice endogenous genes verified the stability, efficiency and ease use of system in rice. At the same time, the rice-amylose synthesis gene OsGBSS down regulated by the MIGS system, and its applicability in rice genetic improvement breeding was verified. This interesting, efficient and simple method could be widely used in monocotyledonous plants such as rice.

## Author Contributions

XZ and YZ conceived and designed the experiments. XZ and LZ generated all vectors. LZ, AT, LY, and QL performed the stable transgenic rice and analyzed the plants. XZ, AT, LJ, KD, and JZ performed the RT-PCR, amylose content analysis, and other experiments. YZ and XZ analyzed the data and wrote the paper. All authors read and approved the final manuscript.

## Conflict of Interest Statement

The authors declare that the research was conducted in the absence of any commercial or financial relationships that could be construed as a potential conflict of interest.
